# Data set of competitive and allosteric protein kinase inhibitors confirmed by X-ray crystallography

**DOI:** 10.1016/j.dib.2021.106816

**Published:** 2021-01-30

**Authors:** Huabin Hu, Oliver Laufkötter, Filip Miljković, Jürgen Bajorath

**Affiliations:** Department of Life Science Informatics, B-IT, LIMES Program Unit Chemical Biology and Medicinal Chemistry, Rheinische Friedrich-Wilhelms-Universität, Friedrich-Hirzebruch-Allee 6, D-53115 Bonn, Germany

**Keywords:** Protein kinase inhibitors, Allosteric mechanism of action, ATP competitive inhibitors, X-ray structures, Target annotations

## Abstract

A data set was generated comprising currently available competitive and allosteric human protein kinase inhibitors confirmed by X-ray crystallography. This data set has been used to systematically explore structural relationships between these types of inhibitors with different mechanisms of action. A major finding of this study has been that these different inhibitor types frequently displayed structural relationships and essentially represented a structural continuum [1]. Use of the data set is not limited to the inhibitor-centric exploration of structural relationships. The collection of kinase inhibitors with structurally confirmed distinct mechanisms of action can also be used, for example, to aid in structure-based drug design or the search for new allosteric kinase inhibitors.

## Specifications Table

**Table:** 

Subject	Drug discovery
Specific subject area	Computational analysis of X-ray structures to generate data sets of kinase inhibitors with different mechanisms and explore structural relationships.
Type of data	Table Figure
How data were acquired	Data were acquired from the Protein Data Bank (PDB) [2] and the KLIFS [3], ProfKin [4], ASD [5], and ChEMBL [6] databases, curated, and organized.
Data format	Secondary data Table (consistently formatted)
Parameters for data collection	The following selection criteria were applied for X-ray structures and compound data (only high-confidence activity data were considered [1]): (1) Organism: Human (2) Kinase type: Protein kinase (3) Crystallographic resolution of at least 3.5 Å (4) Molecular weight of at least 250 Da (5) Inhibitors binding exclusively to the ATP site in kinases (competitive inhibitors) or allosteric sites were selected.
Description of data collection	The data were obtained from a combination of different primary database sources specified above.
Data source location	Department of Life Science Informatics, B-IT, University of Bonn, Friedrich-Hirzebruch-Allee 6, d-53,115 Bonn, Germany.
Data accessibility	The data set has bene deposited on the ZENODO open access platform and is freely available via the following link: https://doi.org/10.5281/zenodo.4436775.
Related research article	H. Hu, O. Laufkoetter, F. Miljković, J. Bajorath, Systematic comparison of competitive and allosteric kinase inhibitors reveals common structural characteristics, Eur. J. Med. Chem. 214, 2021, 113206 [1].

## Value of the Data

• At total of 2763 ATP-competitive and 136 allosteric kinase inhibitors were extracted from X-ray structures and additional target annotations were identified on the basis of compound activity data available in ChEMBL. The data set represents a comprehensive collection of kinase inhibitors with structurally confirmed mechanisms and binding modes.
• Based on the data, the distribution of allosteric kinase inhibitors across different binding sites can be studied and templates for site-specific structure-based design of new allosteric inhibitors be selected. Allosteric inhibitors can also be used as reference compounds for computational screening to search for structurally similar yet distinct compounds as new candidate inhibitors.
• In addition, structural relationships between confirmed competitive and allosteric kinase inhibitors can be analyzed in detail to determine related or chemically distinct core structures for compound design. Furthermore, other active compounds containing these core structures can be identified in databases. Their targets might provide insights into possible multi-target activities of kinase inhibitors with different mechanisms of action.

## 1 Data Description

The data set contains a total of 2899 unique inhibitors including 136 allosteric and 2763 orthosteric compounds that were available in X-ray structures of complexes with a total of 231 protein kinases. For each of these inhibitors with experimentally confirmed mechanism of action, the following information is reported: Protein Data Bank identifier (PDB ID), ligand ID, CHEMBL ID for inhibitors (if available), kinase target UniProt ID [7], standard protein kinase abbreviation, kinase group, SMILES [8] representation of the compound, mechanism of action (competitive or allosteric), and SMILES representation of privileged substructures (if available). The data set is provided in standard tab separated values (tsv) format and made freely available as an open access deposition on the ZENODO platform (https://doi.org/10.5281/zenodo.4436775).

**Fig. 1:**
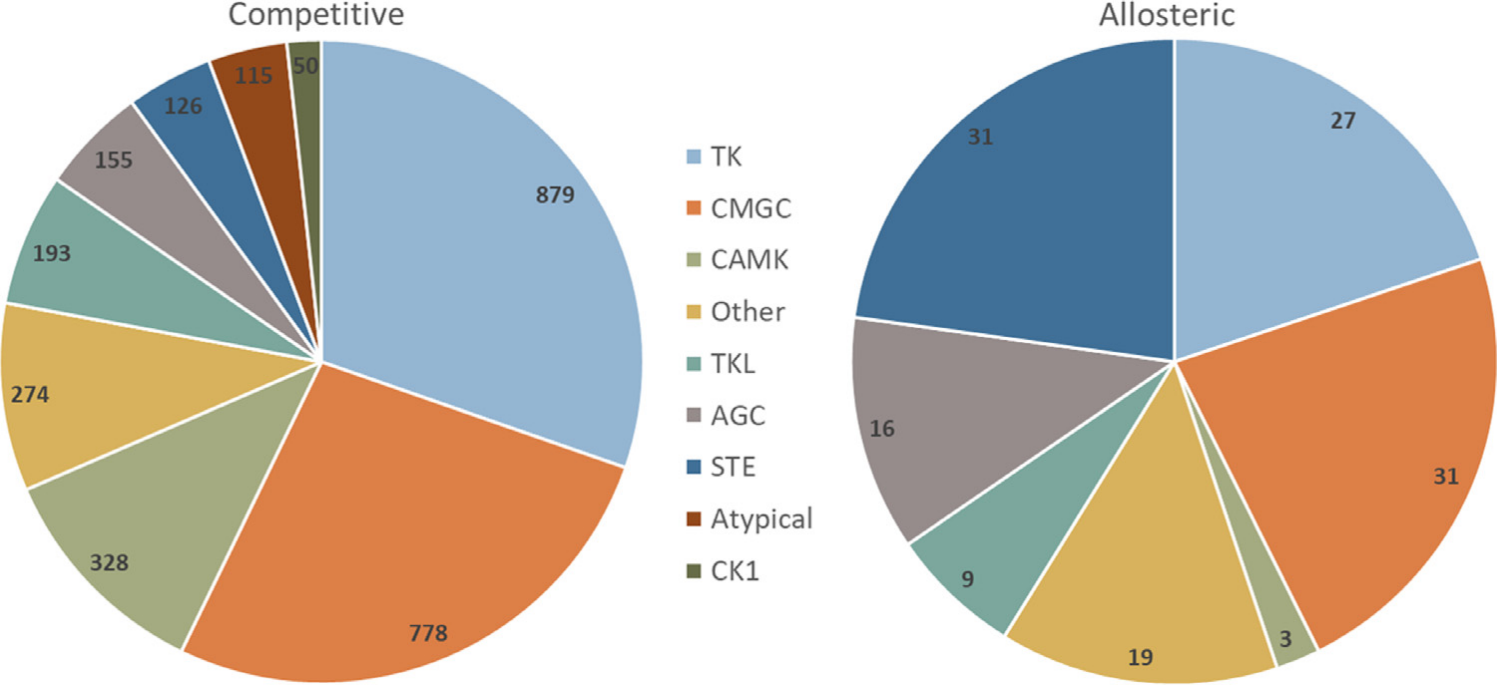
Pie charts show the distribution of competitive (left) and allosteric inhibitors (right) across kinase groups comprising the human kinome. Nine kinase groups are shown and color-coded. For each group, the number of unique inhibitors is reported.

Due to the significant imbalance of the ATP competitive and allosteric inhibitor subsets, it might be assumed that allosteric inhibitors lack representation across the human kinome. However, Fig. 1 shows that the competitive and allosteric inhibitor subsets cover diverse kinase groups. The allosteric subset only lacks inhibitors for two kinase groups (CK1 and Atypical). Most competitive inhibitors are available for the TK, CMGC, and CAMK groups (328–879 unique compounds) while most allosteric inhibitors are available for TK, CMGC, STE (27–31 unique compounds).

**Fig. 2:**
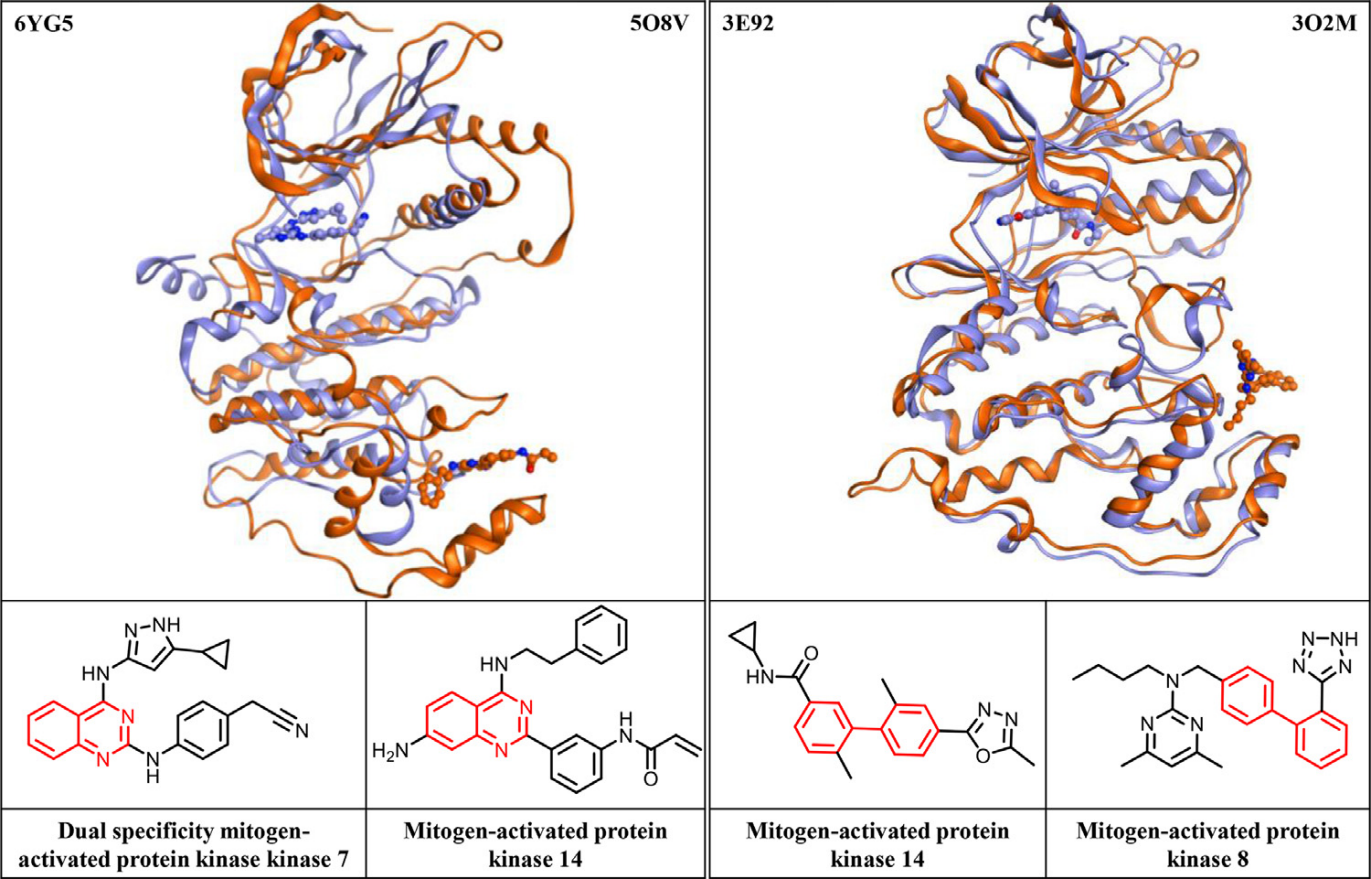
Shown are exemplary competitive and allosteric inhibitors sharing PSs. Corresponding X-ray structures of complexes formed by kinases with competitive or allosteric inhibitors are superimposed and colored in blue and orange, respectively. The PDB IDs of the structures and protein kinase names are provided. PS in inhibitors are colored in red. (For interpretation of the references to color in this figure legend, the reader is referred to the web version of this article.)

A set of privileged substructures (PSs) provided by Welsch et al. [9] was used to examine the occurrence of PSs in kinase inhibitors. PSs are frequently found in compounds active against different target classes. In total, 29 privileged substructures (PSs) were detected in 1022 of the 2899 crystallographic inhibitors (35.2%). These inhibitors were found in structures of complexes with 181 protein kinases. Among the PSs detected in crystallographic inhibitors, indole was most frequently found (in 152 inhibitors), followed by quinoline (137 inhibitors), and phenylpiperazine (126 inhibitors). Three and 10 PSs were exclusively found in allosteric and competitive inhibitors, respectively. Additionally, 16 PSs were identified in both types of inhibitors, hence establishing substructure relationships between allosteric and competitive inhibitors. In Fig. 2, two of these PSs (quinazoline and biphenyl) are shown.

## 2 Experimental Design, Materials and Methods

Allosteric kinase inhibitors were systematically extracted from different databases of X-ray structures including PDB [2], KLIFS [3], ProfKin [4], and ASD [5]. Binding site and binding modes of these inhibitors were confirmed through visual inspection of their structures using the Molecular Operating Environment (MOE) [10]. Competitive kinase inhibitors targeting the ATP site were extracted from KLIFS applying the corresponding selection criterion “Front pocket = 1”. Inhibitors in complex with human protein kinases were retained if their structures had a crystallographic resolution of 3.5 Å or better. Fragment-like compounds with molecular weight < 250 Da were excluded as well as Inhibitors binding to both the ATP and allosteric sites. Substructure search calculations for PSs [9] were carried out with RDKit [11].

## Ethics Statement

This is a secondary data set and thus did not involve any human or animal testing.

## CRediT Author Statement

**Huabin Hu:** Data curation, Data analysis and organization, Writing-Original draft preparation, Writing - Reviewing and Editing; **Oliver Laufkötter:** Data curation, Data analysis and organization, Writing-Original draft preparation, Writing-Reviewing and Editing; **Filip Miljković:** Data analysis and organization, Writing-Original draft preparation, Writing-Reviewing and Editing; **Jürgen Bajorath:** Conceptualization, Supervision, Data analysis, Writing-Original draft preparation, Writing-Reviewing and Editing.

## Declaration of Competing Interest

The authors declare that they have no known competing financial interests or personal relationships which have, or could be perceived to have, influenced the work reported in this article.
